# The use of double lumen cannula for veno-venous ECMO in trauma patients with ARDS

**DOI:** 10.1186/s13049-015-0106-2

**Published:** 2015-03-28

**Authors:** Martin Gothner, Dirk Buchwald, Justus T Strauch, Thomas A Schildhauer, Justyna Swol

**Affiliations:** Department of General and Trauma Surgery, University Hospital Bergmannsheil, Ruhr-University Bochum, Bürkle-de-la-Camp Platz 1, 44789 Bochum, Germany; Department of Cardiothoracic Surgery, University Hospital Bergmannsheil, Ruhr-University, Bochum, Bürkle-de-la-Camp Platz 1, 44789 Bochum, Germany

**Keywords:** ECMO, Trauma, ARDS, Double lumen cannula, Complications

## Abstract

**Background:**

The use of a double lumen cannula for veno-venous extracorporeal membrane oxygenation (v.v. ECMO) offers several advantages such as cannulation with only one cannula, patient comfort and the earlier mobilization and physiotherapy. The cannulation should be performed under visual wire and cannula placement into the right atrium, which is associated with risks of malposition and right ventricular perforation. The aim of this patient series is to describe the use of double lumen cannula in trauma patients with posttraumatic ARDS.

**Material and methods:**

Criteria for the v.v ECMO treatment were defined as hypoxaemia (pO2/FiO2 < 200 mmHg, FiO2 0.8-1,0); tidal volume >4-6 ml/kg ideal body weight; mean inspiratory pressure (Pinsp) >32-34 mmHg; respiratory acidosis pH <7.25; and arterial saturation (SaO2) <90%. The analysis included the Injury Severity Score (ISS), the types of injury, time of treatment, complications and outcomes.

**Results:**

A total of 24 patients with major trauma were treated for posttraumatic ARDS with v.v. ECMO. The double lumen cannula (Avalon®, Fa. Maquet, Rastatt, Germany) was used in six male patients. The mean ISS was 31 (20–48). The ECMO therapy was started in an average on the third day after trauma. The mean ECMO run time was 7 days ± 5 (6–18), and the hospital stay was in mean of 60 days ± 34 (21–105).

**Conclusion:**

The use of double lumen cannula for v.v ECMO therapy in trauma patients is a feasible treatment option. No higher risk of bleeding could be found in this case series. A PTT-controlled heparinization is recommended using double lumen cannula. Therefore the use of this cannula type in trauma patients with high risk of bleeding is to discuss controversially.

## Introduction

The incidence of trauma has consistently decreased over the past few years. In 2005, approximately 196,8 deaths/100,000 population were documented in the US for individuals up to the age of 54 years [1,2]. Young people are especially affected by trauma [3]. The causes of trauma include car accidents, falls and stab injuries [4,5]. Severe trauma is often associated with traumatic thoracic injury, which is reported in up to 50% of cases [6]. Chest trauma can influence the posttraumatic mortality and morbidity rates of trauma patients, and such trauma is directly responsible for death in 25% of all patients with these injuries. Another 25% of all patients with chest injuries die from complications related to other injuries [6,7].

Posttraumatic complications, such as pneumonia, acute lung injury (ALI) or acute respiratory distress syndrome (ARDS), are life-threatening complications that often follow trauma [8-10]. ARDS is a form of lung injury that alters capillary permeability, and the incidence of ARDS in the US is 18-70/ 100.000 inhabitants annually [11-14]. The treatment of ARDS caused by severe chest trauma, trauma to the extremities, or pelvic or spine injuries remains a challenge in intensive and trauma care, and ARDS is often associated with long stays in the intensive care unit [15]. A final recourse for patients with hypoxia that is refractory to conventional therapy modalities and presents with hypercapnia and respiratory acidosis could be treatment with extracorporeal membrane oxygenation (ECMO) [16,17].

The double lumen cannula can be used for veno-venous extracorporeal membrane oxygenation (v.v. ECMO) in patients with acute respiratory distress syndrome (ARDS), as well as in trauma patients or as a bridge to lung transplant [18].

**Figure 1:**
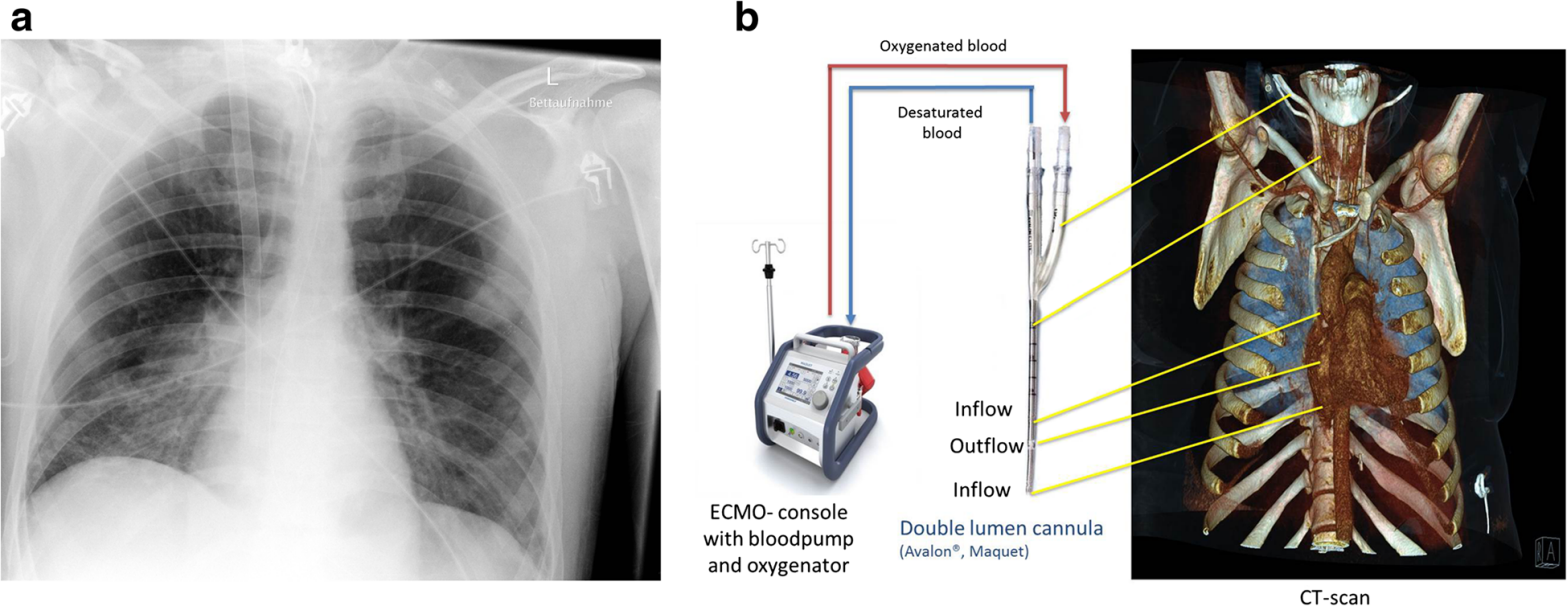
**Chest X-ray and drawing of a double lumen cannula in fulminant ARDS. A**. Chest x-ray 7 days after implementation of a double lumen cannula in the right jugular vein. **B**. Drawing of the technique of a double lumen ECMO with the correct position in the jugular vein, CT Scan-reconstructions.

The cannula consists of 2 lumens. One lumen allows the deoxygenated blood to drain from the distal and proximal ports from the inferior and superior vena cava, while the other lumen allows the oxygenated blood to return from the external oxygenator to the right atrium (Figure 1a and b) [19]. The double lumen cannula is not heparin-coated, and the heparin dosage has to be partial thromboplastin time (pTT) – controlled (pTT 50–60 s). Some trauma patients are in high risk for bleeding or bleeding complications can be fatal for the course like in brain or spinal cord injury [20].

The cannulation with double lumen cannula should be performed under visual fluoroscopic control for the wire. When the cannula passes the right atrium, it is associated with risks of malpositioning of the cannula and right ventricular perforation [19]. Another option for cannulation could be under transthoracic echocardiography, transoesophageal echocardiography, or a combination of transoesphageal echocardiography and fluoroscopic guidance [21,22].

In trauma patients with high risk of bleeding the use of a double lumen cannula has to be discussed controversially. The double lumen cannula is not heparin coated and therefore pTT-controlled heparinization has to be used.

The aim of this study is to describe the experiences with use of double lumen cannula in trauma patients with posttraumatic (ARDS).

## Material and methods

### The analysis was based on the Bergmannsheil ECMO database of patients with major trauma and subsequent moderate or severe ARDS.

The criteria for the v.v. ECMO treatment were defined as follows [23]:

- hypoxaemia (pO_2_/FiO_2_ < 200 mmHg, FiO_2_ 0.8-1,0) during a ventilation time longer than 8 hours;
- tidal volume > 4–6 ml/kg ideal body weight;
- inspiratory pressure (P_insp_) >32-34 mmHg;
- respiratory acidosis (pH <7.25) or/and
- arterial oxygen saturation < 90%.

All of the patients were mechanical ventilated in pressure controlled BIPAP-Mode® (Biphasic Positive Airway Pressure, Fa. Dräger Medical, Lübeck, Germany). Lung protective ventilation with low tidal volumes (4–6 ml/kg BW) was administered, according to the ARDS Network Study Group. ARDS was defined using the Lung- Injury Score described by Murray et al. [24].

IA 31 French cannulas were used (AVALON ELITE® Bi-Caval Dual Lumen Catheter (MAQUET Cardiopulmonary AG, Rastatt, Germany) in all patients in the case series.

The database included the Injury Severity Score (ISS), type of injuries and oxygenation parameters, time of treatment, complications and outcomes. Heparin was given in five patients using continuously intravenous (aim pTT 50–60 sec.). The average heparin dose was 400 IU/hour. No Antithrombin III (AT III) hast to be substituted. In one minor brain injured patient, heparin was started 2 days post trauma after bleeding control and repeated computed tomography scans. Coagulation parameters where screened every 24 hours, Haemoglobin (Hb) minimum every 8 hours. Transfusions were indicated if Hb were less than 8 g/dl. The enteral nutrition was performed obligatory.

## Results

311 trauma patients were treated during the period from 1.1.2008 to 31.12.2012. A total of 24 patients with major trauma (ISS > 25) were treated for posttraumatic ARDS with v.v. ECMO. In six male patients the cannulation was performed using the double lumen cannula in the right jugular vein under fluoroscopy. The cannulation was performed using the percutaneous technique. All patients fulfilled ARDS criteria. The mean Murray Lung injury Score in the group was at mean 2,5.

The mean age was 45 (31–54) years and the mean ISS was 31 (20–48). Three patients had high energy impact car accidents, one patient had a motor bicycle accident, and two patients fell from heights >3 meters. Five patients presented chest trauma with lung contusions, and 2 patients presented severe spine injuries with cervical tetraplegia. One patient suffered a minor brain injury. This patient also presented traumatic amputation of the thigh [Table 1]. Two patients with spinal cord injury had incomplete neurological symptoms and recover far-reaching the walking ability with remaining discrete sensoric disability.

**Table 1:** **Outcomes, complications and in -hospital data of the patients**

**Patient number**	**Pre- ECMO (d)**	**ECMO run (d)**	**Complications**	**ICU LOS (d)**	**Hospital LOS (d)**	**Outcome**	**Transfusion of RBCP**
1	2	15	ARF,VAP	30	40	Alive	29
2	0	7	Bleeding urethra, VAP	13	21	Alive	2
3	3	7	Thrombosis around jugular cannula	18	29	Alive	4
4	13	6	VAP	24	105	Alive	7
5	3	18	VAP	24	85	Alive	4
6	4	7	VAP	16	80	Alive	2
Mean ± SD (min-max)	3 ± 5 (0–13)	7 ± 5 (6–18)		21 ± 7 (13–30)	60 ± 34 (21–105)		8 (2–29)

The data are outlined with means, ARF acute renal failure, VAP ventilator associated pneumonia, SD Standard deviation, (d) days, LOS length of stay, RBCP red blood cell package (300 ml).

The ECMO was implanted on the third day after trauma in average. The average stay in the intensive care unit was 21 days ± 7 (13–30). The mean ECMO run time was 7 days ± 5 (6–18), and the mean hospital stay was 60 days ± 34 (21–105). All of the patients were weaned from ECMO and survived, discharched from the hospital. Thrombosis around and inside double lumen cannula is to mention as a complication, despite the patient received continuously intravenous heparin. Surgical removal of the cannula was necessary. One patient showed bleeding of the urethra, which was self-limiting. One patient developed acute renal failure, for which a continuous haemodiafiltration therapy was necessary for 14 days. Five patients developed ventilator associated pneumonia, which was treated successful with antibiotics. During their stays at the hospital, a mean of 8 red blood cell concentrates (300 ml/unit) had to be substituted.

## Discussion

### Impact of chest trauma

High impact trauma, such as motor vehicle or car accidents, is the most common causes of thoracic injuries [4,25-27]. Complications, such as acute respiratory distress syndrome (ARDS), following severe trauma are associated with high mortality, and for patients with fulminant ARDS, mortality rates of 30%- 50% have been described [14,28]. Some authors have reported high complication rates because of ARDS and multi-organ failure after chest injuries [29]. The implementation of the recommendations of the ARDS Network Study Group, i.e., mechanical ventilation with low tidal volumes (4–6 ml/kgBW) and high positive end-expiratory pressure (PEEP >8) is expected to have protective effects on the lungs and permissive hypercapnia can be tolerated. Extracorporeal membrane oxygenation (ECMO) or extracorporeal lung assistance can be used for ARDS treatment, and survival rates of up to 50 % have been described [30]. The criteria for ECMO treatment are severe hypoxaemia, reduced total thoracic compliance and bilateral infiltrates on chest radiographs.

In 2008, Leone et al. described the long-term outcomes for chest trauma survivors. ARDS developed in 14% of these patients, and 56% of the patients were mechanically ventilated. Their length of stay in the ICU was 4–17 days [31].This group of 105 multiple trauma patients with blunt chest trauma (AIS > 2), who were admitted to the ICU, were evaluated after 6 and 12 months.

### ECMO in trauma, course and complications

This study was a retrospective analysis, based on ELSO -registry data and the Bergmannsheil ECMO database. Severe ARDS with subsequent ECMO therapy is associated with long stays in the ICU, and in this study, the ECMO was implanted at an average of 3 days post-trauma, and the stay at intensive care unit was an average of 21 days (13–30), comparable to other studies. The mean ECMO duration was 7 days (6–18) and the mean hospital stay was 60 days (21–105).

Complications such as renal failure, disseminated intravascular coagulation or clotting of the system have been described [32,33]. In this study, acute renal failure was found in one patient, in whom an intermittent haemodialysis was necessary, comparable to other published studies [34].

Ventilator -associated pneumonia (VAP) is a commonly found complication in associated trauma and with mechanical ventilation [35]. It was found in five patients and was treated successfully with antibiotics. Evans et al. described in their study an association between the length of stay in the hospital and post-traumatic VAP. The mean time in the hospital ranged in their study from 11 to 32 days [35]. In this study, the hospital stays ranged from 21 to 105 days. Those patients with VAP had longer stays in the hospital compared to one patient without VAP.

Using the double lumen cannula is more comfortable for the patient and easier for the nursing. Complications can occur during cannulation as right ventricular rupture with possible subsequent acute cardiac tamponade [19]. Therefore the cannulation should be performed under fluoroscopic control [Table 2].

**Table 2:** **pros and cons of double lumen cannula and femoro-jugular cannulation**

**Veno-venous ECMO**	
femoral-jugular cannulation	double lumen cannula
**PROs**	**PROs**
High blood flow 6–7 L/min possible, No fluoroscopy needed for cannulation, Bedside cannulation possible, Heparin free run possible, Suitable for patients with high risk of bleeding	More comfortable for awake patients, Less or no sedation and less pain medication necessary, Fully mobilization, sitting and walking possible
**CONTRAs**	**CONTRAs**
Risk of femoral cannula kinking during mobilization, Less comfortable for patients, More pain medication, eventually sedation necessary	Fluoroscopy recommended for cannulation, less risk of malposition, bed-side cannulation with high risk with echocardiography possible, pTT 50–60 s needed, not suitable for bleeding patients, patients with severe brain injury or high bleeding risk patients, maximal blood flow about 5 L/min with 31 F cannula

### Limitations

Another limitation of the double lumen cannula are the flow limitations, where only an ECMO flow of more or less 4,5 l/min as an upper limit can be used for treatment. To achieve a sufficient blood flow of the ECMO a 31 french cannula in the jugular vein normally has to be used. The maximal achieved blood flow on ECMO using 31 French double lumen cannula was about 5 L/min. In comparison, using veno-venous femoral-jugular cannulas (e.g. 23–21 F) the maximal blood flow of 6–7 L/min can be achieved [Table 2].

### Balancing between bleeding risk and need for heparin

Bleeding, especially in trauma patients, are among the major complications during ECMO therapy, and they require a special anticoagulation management [36]. Heparin -coated ECMO systems have resulted in decreased haemorrhagic complications [37,38]. As reported by Müller et al. in 2009, miniaturised ECMO systems can decrease haemorrhagic complications, making their implementation possible in trauma patients at high risk for bleeding [39]. Muellenbach et al. reported in 2012 prolonged heparin -free ECMO therapy in multiple injured patients with traumatic brain injuries and intracranial bleeding [33]. They reported in their case series no ECMO -associated bleeding or clotting of the ECMO circuit, and all of the patients survived [33]. Using the single lumen technique, in which heparin -coated cannulas are available; a lower systemic anticoagulation regime is possible. As reported by Ried et al. in 12% of 52 patients developed bleeding complications, which could be treated conservatively [40].

Firstenberg et al. published in 2012 a case report of a 27 -year old man with a fulminant ARDS following severe trauma with traumatic brain injury and intraventricular haemorrhage, as well as subdural haematoma. ECMO therapy was performed, and systemic anticoagulation with heparin was started 48 hours after the trauma. No thrombus formation or clotting complications of the ECMO system is described [20]. Another goal of treatment might be to avoid hypothermia and acidosis with subsequent coagulopathy after trauma [41]. In this study, continuous intravenous heparinisation was performed with a PTT of 50–60 seconds, controlled every 8 hours. One thrombosis around and inside the cannula was observed, and surgical removal of the cannula was necessary to avoid life -threatening complications. This complication has not been previously described.

### Indication for vv ECMO in trauma and outcome

The majority of traumatic pulmonary and chest injuries can be managed without vv ECMO. However vv ECMO offer supplemental capacity in the treatment of secondary complications of patients with major injuries when their primary injuries are being evaluated and treated. ECMO should be considered in trauma patients in acute severe respiratory failure and/or injury of the trachea-bronchial tree, resulting in inadequate gas exchange. The current treatment of ARDS has been improved, which is demonstrable by use of ECMO. The CESAR study, the only major randomized controlled trial on ECMO in ARDS, showed that the transfer of adult patients with severe but potentially reversible respiratory failure to a center with an ECMO-based management protocol significantly improves survival without severe disability [23]. Twelve trauma patients were included in the CESAR study [23].

Despite several cohort studies without control group the evidence for the use of vv ECMO in this setting is not supported by a randomized trial. Therefore, if vv ECMO is introduced as a routine program (inclusive policies and team training) it starts with lives being saved.

Vv ECMO is independently associated with survival in adult trauma patients with respiratory failure. A cohort of 17 ECMO/ECLS and 17 conventional machine ventilated patients, matched for age and lung injury severity, also had significantly greater survival in the ECMO/ECLS group [42]. In conjunction with the appropriate consideration of physiology, knowledge from the bedside and information generated from observational studies, the appropriate use of objective research on ECMO/ECLS evidence will continue to play a major role in its future development (Table 3).

**Table 3:** **Cohort studies “ECMO/ECLS in Trauma” (without control group)**

**Author, Publication year**	**Number of patients**	**Survival**	**Citation**
Anderson 1994	24	17 weaned, 15 discharged	[43]
Senunas 1997	14	8 survivors	[44]
Michaels 1999	30	17 weaned 15 discharged	[30]
Cordell-Smith 2006	28	20 survivors	[45]
Huang 2009	9	7 survivors	[37]
Arlt 2010	10	6 survived	[46]
Ried 2013	52	79 % survived	[40]
Biderman 2013	10	7 survivors	[47]
Bonacchi 2013	14	5 survivors	[48]
Tseng 2014	9	7 weaned, 3 survived	[49]
Wu 2014	20	16 survivors	[50]

## Conclusion

The use of a double lumen cannula in v.v. ECMO therapy in trauma patients with a post-traumatic ARDS is a feasible treatment option. Rapid reduction of sedation, early mobilization is possible. Further studies have to be performed to underline these possible positive effects. We do not recommend the double lumen cannula in patients with high risk of the bleeding or in hemorrhagic shock because a pTT controlled heparinization is recommended.

## Abbreviations

ALI: Acute lung injury
ARDS: Acute Respiratory Distress Syndrome
BHL: Bergmannsheil University Hospital Bochum, Germany
BMI: Body Mass Index
CPR: Cardiopulmonary resuscitation
CRRT: Continuous renal replacement therapy
ECD: Extracorporeal devices
ECLS: Extracorporeal Life Support
ECPR: ECLS during CPR
ECMO: Extracorporeal Membrane Oxygenation
ICU: Intensive Care Unit
CT: Computer tomography
MV: Mechanical ventilation
LOS: Length of stay
pECLA: Pumpless extracorporeal lung assist
P/F pO_2_/FiO_2_: Ratio
PRBC: Packed red blood cells
v.v.: Veno-venous
v.a.: Veno-arterial

## References

[1] Huber-Wagner S, Stegmaier J, Mathonia P, Paffrath T, Euler E, Mutschler W (2010). The sequential trauma score - a new instrument for the sequential mortality prediction in major trauma. Eur J Med Res.

[2] Stoll MC, Rademacher F, Klak K, Strauch J, Schildhauer TA, Swol J (2014). Veno-venous extracorporeal membrane oxygenation therapy of a severely injured patient after secondary survey. Am J Emerg Med.

[3] Geyer LL, Korner M, Linsenmaier U, Wirth S, Reiser M, Meindl T. The role of follow-up ultrasound and clinical parameters after abdominal MDCT in patients with multiple trauma. Acta radiologica. 2013. doi:10.1177/0284185113499559 10.1177/0284185113499559 23969264

[4] Kiraly L, Schreiber M (2010). Management of the crushed chest. Crit Care Med.

[5] Pettiford BL, Luketich JD, Landreneau RJ (2007). The management of flail chest. Thorac Surg Clin.

[6] Stahel PF, Schneider P, Buhr HJ, Kruschewski M (2005). [Emergency management of thoracic trauma]. Orthopade.

[7] Knoferl MW, Liener UC, Seitz DH, Perl M, Bruckner UB, Kinzl L (2003). Cardiopulmonary, histological, and inflammatory alterations after lung contusion in a novel mouse model of blunt chest trauma. Shock.

[8] Gothner M, Buchwald D, Schlebes A, Strauch JT, Schildhauer TA, Swol J (2013). Use of extracorporeal membrane oxygenation in combination with high-frequency oscillatory ventilation in post-traumatic ARDS. Acta Anaesthesiol Scand.

[9] Havlicek K, Motycka V, Siller J, Cervinka V (2005). Systemic inflammatory response syndrome (SIRS) in serious chest injuries: is a pharmacological blockade effective?. Ann Thorac Cardiovasc Surg.

[10] Waydhas C, Nast-Kolb D (2006). Chest injury. Part I: Significance–symptoms–diagnostic procedures. Unfallchirurg.

[11] Lewandowski K (2000). Extracorporeal membrane oxygenation for severe acute respiratory failure. Crit Care.

[12] Lewandowski K, Metz J, Deutschmann C, Preiss H, Kuhlen R, Artigas A (1995). Incidence, severity, and mortality of acute respiratory failure in Berlin, Germany. Am J Respir Crit Care Med.

[13] Luce JM (1998). Acute lung injury and the acute respiratory distress syndrome. Crit Care Med.

[14] Suchyta MR, Clemmer TP, Orme JF, Morris AH, Elliott CG (1991). Increased survival of ARDS patients with severe hypoxemia (ECMO criteria). Chest.

[15] Bamvita JM, Bergeron E, Lavoie A, Ratte S, Clas D (2007). The impact of premorbid conditions on temporal pattern and location of adult blunt trauma hospital deaths. J Trauma.

[16] Keel M, Meier C (2007). Chest injuries - what is new?. Curr Opin Crit Care.

[17] Keel M, Trentz O (2005). Pathophysiology of polytrauma. Injury.

[18] Reeb J, Falcoz PE, Santelmo N, Massard G (2012). Double lumen bi-cava cannula for veno-venous extracorporeal membrane oxygenation as bridge to lung transplantation in non-intubated patient. Interact Cardiovasc Thorac Surg.

[19] Hirose H, Yamane K, Marhefka G, Cavarocchi N (2012). Right ventricular rupture and tamponade caused by malposition of the Avalon cannula for venovenous extracorporeal membrane oxygenation. J Cardiothorac Surg.

[20] Firstenberg MS, Nelson K, Abel E, McGregor J, Eiferman D (2012). Extracorporeal membrane oxygenation for complex multiorgan system trauma. Case Rep Surg.

[21] Chimot L, Marque S, Gros A, Gacouin A, Lavoue S, Camus C (2013). Avalon(c) bicaval dual-lumen cannula for venovenous extracorporeal membrane oxygenation: survey of cannula use in France. ASAIO J.

[22] Abrams D, Brodie D, Javidfar J, Brenner K, Wang D, Zwischenberger J (2012). Insertion of bicaval dual-lumen cannula via the left internal jugular vein for extracorporeal membrane oxygenation. ASAIO J.

[23] Peek GJ, Mugford M, Tiruvoipati R, Wilson A, Allen E, Thalanany MM (2009). Efficacy and economic assessment of conventional ventilatory support versus extracorporeal membrane oxygenation for severe adult respiratory failure (CESAR): a multicentre randomised controlled trial. Lancet.

[24] Murray JF, Matthay MA, Luce JM, Flick MR (1988). An expanded definition of the adult respiratory distress syndrome. Am Rev Respir Dis.

[25] Boyd AD, Glassman LR (1997). Trauma to the lung. Chest Surg Clin N Am.

[26] Shorr RM, Crittenden M, Indeck M, Hartunian SL, Rodriguez A (1987). Blunt thoracic trauma. Analysis of 515 patients. Ann Surg.

[27] Trupka A, Nast-Kolb D, Schweiberer L (1998). Thoracic trauma. Unfallchirurg.

[28] Schuerer DJ, Kolovos NS, Boyd KV, Coopersmith CM (2008). Extracorporeal membrane oxygenation: current clinical practice, coding, and reimbursement. Chest.

[29] Johnson JA, Cogbill TH, Winga ER (1986). Determinants of outcome after pulmonary contusion. J Trauma.

[30] Michaels AJ, Schriener RJ, Kolla S, Awad SS, Rich PB, Reickert C (1999). Extracorporeal life support in pulmonary failure after trauma. J Trauma.

[31] Leone M, Bregeon F, Antonini F, Chaumoitre K, Charvet A, Ban LH (2008). Long-term outcome in chest trauma. Anesthesiology.

[32] Gorlinger K, Bergmann L, Dirkmann D (2012). Coagulation management in patients undergoing mechanical circulatory support. Best Pract Res Clin Anaesthesiol.

[33] Muellenbach RM, Kredel M, Kunze E, Kranke P, Kuestermann J, Brack A (2012). Prolonged heparin-free extracorporeal membrane oxygenation in multiple injured acute respiratory distress syndrome patients with traumatic brain injury. J Trauma Acute Care Surg.

[34] Jamal JA, Economou CJ, Lipman J, Roberts JA (2012). Improving antibiotic dosing in special situations in the ICU: burns, renal replacement therapy and extracorporeal membrane oxygenation. Curr Opin Crit Care.

[35] Evans HL, Warner K, Bulger EM, Sharar SR, Maier RV, Cuschieri J (2011). Pre-hospital intubation factors and pneumonia in trauma patients. Surg Infect (Larchmt).

[36] Voelckel W, Wenzel V, Rieger M, Antretter H, Padosch S, Schobersberger W (1998). Temporary extracorporeal membrane oxygenation in the treatment of acute traumatic lung injury. Can J Anaesth.

[37] Huang YK, Liu KS, Lu MS, Wu MY, Tsai FC, Lin PJ (2009). Extracorporeal life support in post-traumatic respiratory distress patients. Resuscitation.

[38] Madershahian N, Wittwer T, Strauch J, Franke UF, Wippermann J, Kaluza M (2007). Application of ECMO in multitrauma patients with ARDS as rescue therapy. J Card Surg.

[39] Muller T, Philipp A, Luchner A, Karagiannidis C, Bein T, Hilker M (2009). A new miniaturized system for extracorporeal membrane oxygenation in adult respiratory failure. Crit Care.

[40] Ried M, Bein T, Philipp A, Muller T, Graf B, Schmid C (2013). Extracorporeal lung support in trauma patients with severe chest injury and acute lung failure: a 10-year institutional experience. Crit Care.

[41] Saxena P, Shehatha J, Boyt A, Newman M, Konstantinov IE (2009). Role of extracorporeal circulation in the management of accidental deep hypothermia. Heart Lung Circ.

[42] Guirand DM, Okoye OT, Schmidt BS, Mansfield NJ, Aden JK, Martin RS (2014). Venovenous extracorporeal life support improves survival in adult trauma patients with acute hypoxemic respiratory failure: a multicenter retrospective cohort study. J Trauma Acute Care Surg.

[43] Anderson HL, Shapiro MB, Delius RE, Steimle CN, Chapman RA, Bartlett RH (1994). Extracorporeal life support for respiratory failure after multiple trauma. J Trauma.

[44] Senunas LE, Goulet JA, Greenfield ML, Bartlett RH. Extracorporeal life support for patients with significant orthopaedic trauma. Clinical orthopaedics and related research. 1997(339):32-40. 10.1097/00003086-199706000-00005 9186198

[45] Cordell-Smith JA, Roberts N, Peek GJ, Firmin RK (2006). Traumatic lung injury treated by extracorporeal membrane oxygenation (ECMO). Injury.

[46] Arlt M, Philipp A, Voelkel S, Rupprecht L, Mueller T, Hilker M (2010). Extracorporeal membrane oxygenation in severe trauma patients with bleeding shock. Resuscitation.

[47] Biderman P, Einav S, Fainblut M, Stein M, Singer P, Medalion B (2013). Extracorporeal life support in patients with multiple injuries and severe respiratory failure: a single-center experience?. J Trauma Acute Care Surg.

[48] Bonacchi M, Spina R, Torracchi L, Harmelin G, Sani G, Peris A (2013). Extracorporeal life support in patients with severe trauma: an advanced treatment strategy for refractory clinical settings. J Thorac Cardiovasc Surg.

[49] Tseng YH, Wu TI, Liu YC, Lin PJ, Wu MY (2014). Venoarterial extracorporeal life support in post-traumatic shock and cardiac arrest: lessons learned. Scand J Trauma Resusc Emerg Med.

[50] Wu MY, Lin PJ, Tseng YH, Kao KC, Hsiao HL, Huang CC (2014). Venovenous extracorporeal life support for posttraumatic respiratory distress syndrome in adults: the risk of major hemorrhages. Scand J Trauma Resusc Emerg Med.

